# An Observational Cohort of First Episode Psychosis in Iran: The Azeri Recent Onset Acute Phase Psychosis Survey (ARAS Cohort) Study Protocol

**DOI:** 10.3389/fpsyt.2021.627960

**Published:** 2021-02-10

**Authors:** Sara Farhang, Mehrdad Ghaemmaghami, Ali Reza Shafiee-Kandjani, Seyed Gholamreza Noorazar, Wim Veling, Ayyoub Malek, Mohammad Hossein Somi, Richard Bruggeman, Behrooz Z. Alizadeh

**Affiliations:** ^1^University Medical Center Groningen, University Center for Psychiatry, Rob Giel Research Center, University of Groningen, Groningen, Netherlands; ^2^Research Center of Psychiatry and Behavioral Sciences, Tabriz University of Medical Sciences, Tabriz, Iran; ^3^Faculty of Medicine, Tabriz University of Medical Sciences, Tabriz, Iran; ^4^Department of Psychiatry, University Medical Center Groningen, University of Groningen, Groningen, Netherlands; ^5^Liver and Gastrointestinal Diseases Research Center, Tabriz University of Medical Sciences, Tabriz, Iran; ^6^Department of Epidemiology, University Medical Center Groningen, University of Groningen, Groningen, Netherlands

**Keywords:** schizophrenia, first episode psychosis (FEP), social context, cognition, gene-environement interactions

## Abstract

**Background:** Most of our knowledge about the etiology, course, treatment, and outcome of schizophrenia spectrum and other psychotic disorders stems from Western countries. Data from populations living in other geographical areas and low- and middle-income countries, with different genomes (ethnicity) and exposomes (e.g., culture and social support, drugs of abuse, religion), will add to our knowledge of this complex disorder.

**Methods:** The Azeri Acute phase/Recent onset psychosis Survey (ARAS) has been initiated to study the course of the disorder in patients with recent-onset psychosis using validated diagnostic tools and a comprehensive outcome monitoring system, aiming to reveal indicators for understanding the risk and resilience factors and for choosing the best-personalized treatment strategy. All participants will be evaluated for clinical signs and symptoms as well as risk and resilience factors and will be followed up for 1, 3, and 5 years for outcomes in several domains. A hierarchical cluster method will be applied to identify the number of clusters for each outcome. Defined models will be applied to assess the predictive value of cognition on symptomatic and functional outcomes at follow-up.

**Discussion:** The ARAS cohort will yield significant academic- (research and education) and care-related achievements. ARAS data and experience will have value both in being a useful model for other parts of this region and in an expansion of the currently available knowledge.

## Background

Schizophrenia, as the prototype of the schizophrenia spectrum (SSD) and other psychotic disorders (1), remains one of the most costly disorders in terms of human suffering and societal expenditure (2, 3). It contributed 1.7% of total years of life lived with disability (YLDs) to the global burden of disease in 2016. The incidence of schizophrenia varies across and within countries, as a wide distribution range is noticeable in available studies (4, 5). A systematic review of 161 studies reported a median incidence rate of 15.2 per 100,000, with significant effects of migrant status, gender, and urbanicity (6, 7). There have also been differences noted in point and lifetime prevalence estimates related to economic status and latitude (8, 9). The course of illness may also differ across contexts. Older studies suggested that the course of psychotic disorders may be more favorable in low- and middle-income countries (LMIC) (WHO 10 country study), while other studies point at a large treatment gap as a cause of poor outcome in LMIC (10). Socio-cultural differences such as social support, religious and cultural beliefs, and differences in mental health care organization might explain a substantial portion of these epidemiological variations (11).

SSDs and other psychotic disorders are characterized by positive symptoms including hallucinations and delusions, negative symptoms such as apathy and social withdrawal, and cognitive impairment. Somatic comorbidity shows a high prevalence, of which the most common types include metabolic syndrome, diabetes mellitus, and cardiovascular diseases (12), adding to a remarkable increase in the rate of all-cause mortality in recent decades (9).

SSDs are considered complex genetic disorders. The heterogeneous clinical presentation most likely results from a variety of gene-environment interactions (13). A wide body of evidence suggests that highly dynamic multi-dimensional interactions between small effects of the genome and exposome underlie the etiopathogenesis of SSDs (13, 14). The exposome encompasses environmental factors, such as social, physical, chemical, biological, and behavioral patterns, over a lifetime, which can all vary widely between populations, in a similar manner to the genome (15).

Antipsychotic medication, cognitive-behavioral therapy, and rehabilitation are the cornerstones of treatment. Antipsychotic medications, the underlying illness, and their interaction increase the risk of cardiometabolic abnormalities in patients with SSD (16–18). Here, lifestyle and diet affect this process, and, again, these might differ substantially between populations.

Of note, incomplete response to pharmacotherapy is seen in 30–40% of patients (19). This proportion has remained almost unchanged over time, despite several decades of pharmacological improvements, and the discovery of new medications (20). Meanwhile, studies continue to examine indicators for a better response to a specific medication or liability for side effects (21). However, the predictive value of this personalized approach still needs to be increased before it can be applied to clinical practice.

Despite the differences in genomes, exposomes, and lifestyles across populations, the main volume of the current knowledge about risk and resilience factors for SSD has been collected in high-income Western countries. Recently, some studies from Asia have enriched the current knowledge concerning the role of diverse ethnicity and different living situations on SSD (22, 23). Beyond ethnicity, factors such as childhood adversity, trauma, use of illicit drugs, neurocognitive abilities, social and familial support, and genetic factors all constitute well-established modulators of SSD. So far, there are very limited data on SSD from the Middle East and Central Asia, including Iran, and specifically from populations with Turkish heritage. Thus, little is known about the attribution of environmental factors and genetics to the development of SSD in Middle Eastern and Central Asian populations. Given differences in genetics, culture, lifestyle, and population beliefs in these middle- and low-income countries, such as Iran, it is expected that risk and resilience factors, the magnitude of their interaction, as well as the course of SSD are different from those of Western, high-income countries. Investigating these differences between populations and their roles in the development and course of SSD provides a unique opportunity to understand the full spectrum of factors involved in the etiology and pathogenesis of SSD and other psychotic disorders.

Diagnostic procedures, treatment, and rehabilitation in Iran are based on well-known international diagnostic classification systems and up-to-date western guidelines (24). Nevertheless, serious gaps in in-depth research with a focus on the causes, management, and consequences of SSD exist in this population (10, 25, 26). Looking at Iran as an example of a naturally rich, but low-income country, we previously observed patients with SSD and their families facing a variety of social, familial, and economic challenges (27, 28).

Accurate knowledge on the burden of SSD on the personal, societal, and health care systems is lacking, as comprehensive national registry data are not yet available. For the same reason, little is known about the outcome of Iranian patients with SSD. It can be argued that the “real-world' implementation of evidence-based practices will be hampered by social and economic factors resulting in sub-optimal care of Iranian patients. Therefore, it is essential to establish well-designed sufficiently large longitudinal studies to investigate the risk and resilience factors involved in SSD in Middle East regions. We, therefore, initiated the Azeri Recent onset/Acute phase psychosis Survey (ARAS), which investigates the course of recent-onset psychosis by using validated diagnostic tools, setting up a comprehensive outcome monitoring system, aiming to reveal indicators for understanding the risk and resilience factors associated with SSD, and for choosing the best-personalized treatment strategy. Next, to improve mental health care, the ARAS-project will define and validate the first population-specific guideline for the management of SSD in Iran. Participants will be followed up at years 1, 3, and 5 after recruitment by face to face interview using structured validated questionnaires. The specific objectives of this study are to (I) determine the disorder course regarding clinical and social aspects and predefined its subtypes, (II) identify genetic and environmental risk and resilience factors, (III) investigate whether SSD subtypes of clinical and social aspects can predict patient prognosis, and (IV) integrate findings from objectives I to III to build patient-centered management of psychosis.

## Methods

### Study Population

The catchment area for this observational cohort study is East Azerbaijan, a province in the North of Iran with more than 3, 7 mln inhabitants, with the majority having an Azeri ethnic background. Iranian Azeris are the largest minority in the country with a Caucasian ethnic background This community is the world's largest Azeri population who are a Turkic ethnic group. The dominant religion in this region is Islam, followed by Christianity, Zartosht, and Jewish.

### Eligibility Criteria

The study target population includes all inhabitants of East Azerbaijan province who are referred with (signs of) a first psychotic episode or evaluated for a recurrent psychotic episode that was previously undiagnosed (<2 years). The cohort will include patients with a diagnosis of schizophrenia, schizophreniform disorder, delusional disorder, brief psychotic disorder, schizotypal personality disorder, schizoaffective disorder, or substance-induced psychotic disorder based on the Diagnostic and Statistical Manual of Mental Disorders (DSM)-5 (1). Patients receiving any of these diagnoses within the 2 years prior to the inclusion date, will be invited to participate in the study. A patient will be included only after giving written informed consent. If a patient is mentally incapable of giving informed consent, as considered by the study clinicians, this patient will be either excluded or the patient's first-degree relatives or attorney will be requested to sign the informed consent. There will be no other exclusion criteria.

### Study Set-Up

The ARAS project was approved by the ethical committee of the National Institute for Medical Research Development (NIMAD) in Iran in 2017 (record number: IR.NIMAD.REC.1396.101). All study procedures will be carried out according to the Declaration of Helsinki. Written informed consent will be obtained from all participants and their caregivers or legal guardians. Patients can leave the study at any time for any reason with no influence on their quality of care. A code will be given to each patient and all data will be recorded anonymously. The participants will be informed that all study findings will be stored and handled in strict confidence according to national guidelines. The principal investigator and the main investigators have access to the materials.

There have been several meetings with psychiatrists and psychologists all over the province to describe the aim of this study. With the incentive of a free, standard, and comprehensive evaluation of their patients, they were invited to refer their patients for participation. Reports are sent back to the referring colleague in all of the sessions along with any suggestion for additional workups. Travel costs are reimbursed for patients, and waiting room refreshments were provided during assessments.

**Figure 1:**
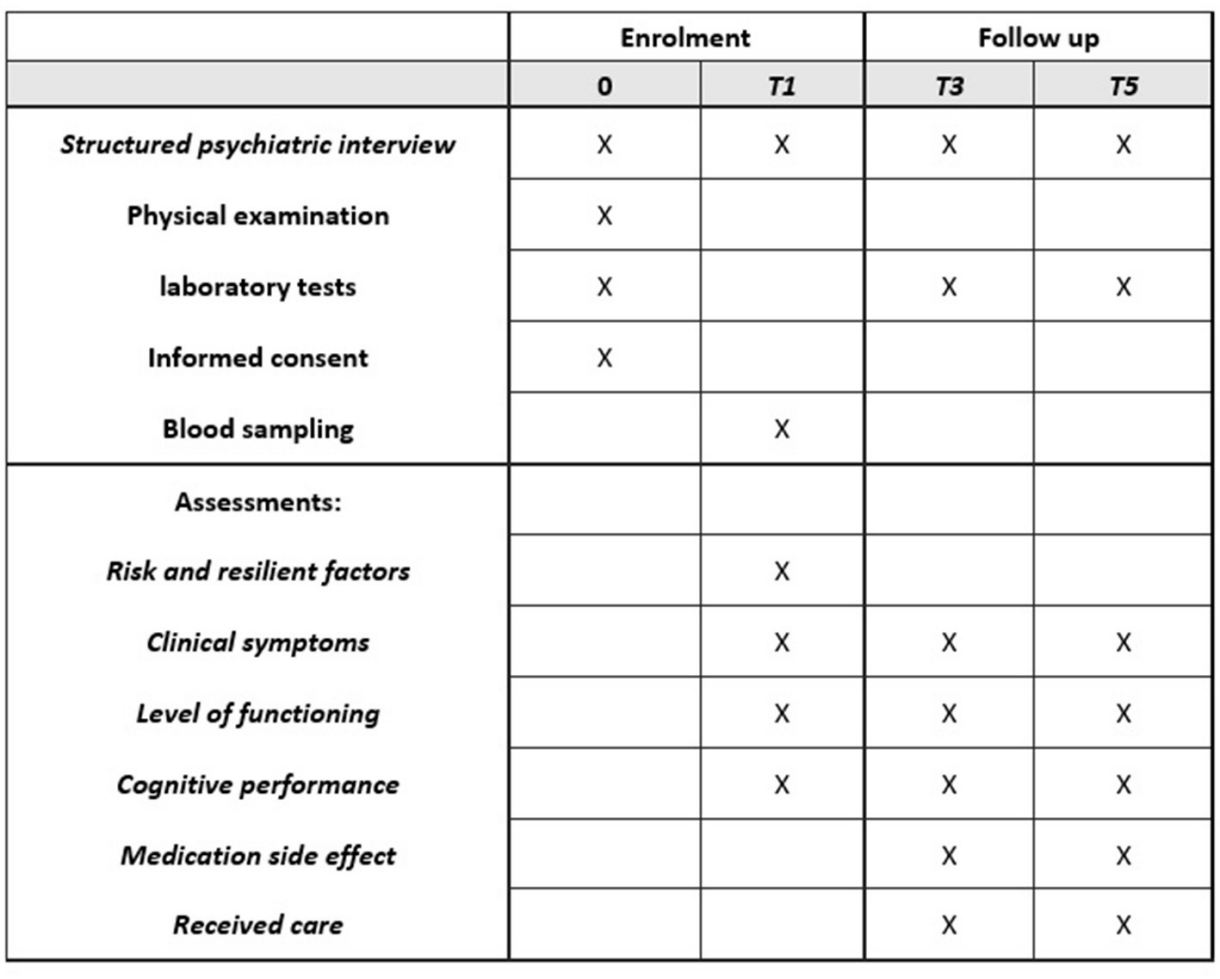
The schedule of enrolment, assessments and follow-up in the ARAS study.

This project started in January 2018. For the first baseline assessment (referred to as T0, Figure 1), clinical interviews and assessments will take place at the treating clinics, starting at the time of admission or as soon as possible if forced admission is required. Psychological testing will be performed within 1 month after inclusion. Diagnostic interviews, questionnaires, and other instruments will be conducted in Farsi, the official language of the country.

Upcoming assessment as routine outcome monitoring will be scheduled for all patients at the 1 year follow up (T1), which will include the same assessments used in T0, including outcome measurements and assessment for direct costs. The second follow-up (T3) is scheduled at 3 years and the final follow-up assessment at 5 years (T5) after inclusion. Treatment strategy and visit intervals will be defined by the treating psychiatrist between inclusion and follow-up periods.

### Assessments

Assessments will be performed in separate meetings depending on their preferred schedule (Figure 1). Clinical assessments will be performed by test psychologists/psychiatrists who are trained by repeated workshops.

#### Clinical Diagnosis and Biography

Clinical diagnoses will be made by referring psychiatrists from in- and out-patient clinics. The clinical diagnosis within the SSD will be confirmed by the Structured Clinical Interview for DSM-5 (SCID) (29), or for patients <18 years by the Kiddie-Schedule for Affective Disorders and Schizophrenia Present and Lifetime Versions (K-SADS-PL) (30). Farsi versions of these two questionnaires have been used by several research teams inside Iran and contain few cultural adaptations (31). In terms of a discrepancy between the diagnosis made by the treating clinician and researcher-administered interview, athird expert opinion from an independent psychiatrist is requested. For special cases, a panel of experts will discuss the patients' profiles to arrive at a consensus. The referring clinician will be informed about the outcome and if the patient will be included.

Dimensions of Psychosis will be rated using the symptom severity dimension tool of DSM-5 (32). The severity of symptoms will be rated using the Positive and Negative Syndrome Scale (PANSS), which is a semi-structured interview on the symptom severity including three subscales of positive and negative symptoms and general psychopathology (33). The severity of depressive symptoms will be measured using the Calgary Depression Scale in Schizophrenia (CDSS) (34). This instrument differentiates between depression and the negative and positive symptoms of schizophrenia (35).

Side effects of antipsychotics and the patients' adherence to their medication will be assessed using the Subject's Response to Antipsychotics (SRA-34) (36). Evaluation for hyperkinesia, parkinsonism, akathisia, and dystonia will be performed using the St. Hans Rating Scale (SHRS) (37).

A medical history will be obtained from the patient and the first-degree relatives of patient. The nature, severity, and consequences of symptoms for daily functioning will be recorded, and, besides their medication-history, nicotine, illicit drug, and alcohol use, and history of illness in the family will be recorded. The biography focuses on signs of an early developmental disorder, current living situation, occupation and daytime activities, and social interactions.

#### Medical Assessments

A physical health status examination, including a physical examination and measurement of blood pressure, heart rate, height, weight, and waist circumference will be carried out. Laboratory tests will be conducted to test for general health conditions, including complete blood cell counts, lipid profiles, fasting blood sugar and glycohemoglobin A1c (HbA1c), liver function tests, kidney function tests, and pituitary function tests, and urine analysis for drugs including morphine, amphetamine, cannabis, tramadol, methadone and presence of abnormal routin tests. When clinically indicated, additional examination will be performed such as brain imaging, electroencephalography, or electrocardiography by the treating psychiatrists. The requirement for external consultation for any general medical condition will be decided by the psychiatrist and results will be recorded.

#### Risk and Resilience Factors

The evaluation process will continue with semi-structured interviews and self-report questionnaires in different domains. History of experienced adversities during early life and peer interaction in primary school will be evaluated using the Illinois Bully Scale (38), Olweus Bully/Victim Questionnaire (39), and the Retrospective bullying experience (40). The Illinois Bully Scale includes items that address how often youth is engaged in social aggression, physical, and verbal types of victimization and physical fighting with peers (34). The Olweus Bully/Victim Questionnaire includes the initiation of an act of bullying against the participant, as well as the expression of bullying behavior against others. The questions include several aspects of bully/victim problems including physical, verbal, indirect, racial, and sexual bullying annoyance. The questionnaire includes pro-bullying and pro-victim attitudes and the reaction of teachers, peers, and parents to the bullying behavior (35). The Retrospective bullying experience addresses the adult population. It measures the frequency, significance, and extent of bully-victimization. Important aspects like bully-related psychological trauma, suicidal ideation if bullied, and bullying in college and the workplace are also addressed (36).

Life events will be recorded using the modified Holmes-Rahe stress scale (41). This inventory includes 50 stressful events adapted to the cultural and social context of the Iranian population that are evaluated by Yes/No questions. The Internalized Stigma of Mental Illness scale (ISMI), a self-report questionnaire measuring self-stigma among persons with psychiatric disorders (42), will also be used. The Multidimensional Scale of Perceived Social Support (43) will evaluate the extent of support from family, friends, or significant people in patients' lives.

Religiosity will be measured using a scale based on Stark and Glock's dimensions of religiosity. This scale is adapted to the Islamic religion measuring dimensions of religious beliefs, practice, experience, and consequences with 26 questions (44).

Assessment of functioning will include (i) WHO Disability Assessment Schedule (WHODAS 2.0) (45), a standard tool applicable to clinical and general populations that covers six dimensions of cognition, mobility, self-care, getting along, life activities, and participation; (41) (ii) Global Assessment of Functioning (GAF) Scale form DSM (1) and (iii) the three-item Functional Recovery Tool including daily living and self-care, work, study, and housekeeping, and social contacts (46, 47). The Manchester Short Assessment of Quality of Life (MANSA) (48) will be used to assess satisfaction with life as a whole and with several domains like leisure, relationships, and mental health (42).

#### Cognitive Assessments

A comprehensive neurocognitive battery has been designed following the recommendations of the Measurement and Treatment Research to Improve Cognition in Schizophrenia (MATRICS) initiative (49), including eight cognitive domains (see Table 1).

**Table 1:** Different domains of data collection at baseline and at 1, 3 and 5 years follow-up.

**Outcome parameter**	**Instrument**
Diagnosis (stability)	Structured psychiatric interview
Symptom severity	Positive and Negative Syndrome Scale, Dimensions of Psychosis symptom severity dimension tool of DSM-5
Depressive symptoms	Calgary Depression Scale in Schizophrenia
Medication side effect	St. Hans Rating Scale, Subjective Response to Antipsychotics-34
Metabolic profile	Laboratory testing, anthropometrics
**Cognitive outcome**	
Working memory	Forward digit span task, Backward digit span task, Letter-number sequencing task, stroop task
Attention	Trail making task
Visual and auditory memory (immediate-delayed), recognition, retention, and learning	Auditory verbal learning test, Rey Osterrieth complex figure
Executive functioning	Symbol digit modality task, letter digit modality task, Verbal fluency task
Speed of processing	Stroop task
Intelligence quotation	Wechsler intelligence scale IV
Social cognition	Benton Facial Recognition Test
Theory of mind	Sali Ann
**Functional outcome**	
Disability	WHO Disability Assessment Schedule
Functional remission	Functional remission tool
Quality of life	Manchester Short Assessment of Quality of Life
Social, occupational, and psychological functioning	Global Assessment of Functioning

Working memory will be tested with the forward and backward digit span and letter-number sequencing tasks (50). Divided attention, which is closely tied to executive functioning (51), will be assessed using the comprehensive (part B) test of the Trail making task. Verbal memory will be tested using The Rey Auditory Verbal Learning Test, measuring memory span, new learning, retention, recognition, and delayed recall (52). Visual learning will be measured using the Rey-Osterrieth Complex Figure. Executive functioning will be evaluated using the symbol digit modality task, letter digit modality task, backward digit span, and letter-number sequencing task. Speed of information processing will be measured with the average score of both the basic (part A) tests of Stroop (53) and Trail making (54), measured as time to complete in seconds, as well as category fluency by naming items from three different categories. The Stroop test will also give an estimation of inhibition and flexibility. Intelligence will be tested using the Wechsler Intelligence Scale-IV short version (55), validated for the Iranian population. Regarding social cognition, emotional recognition will be evaluated using the Reading the mind in the eyes test (56). The Benton Facial Recognition Test will be used to evaluate performance in face discrimination. Theory of mind will be tested using a Sally-Ann story in the Farsi language.

#### Blood Sampling

A trained nurse will collect 10 mL venous blood samples in EDTA (ethylenediaminetetraacetic acid) tubes utilizing a standard venipuncture at the local hospital laboratory. An individual specific barcode for each patient will be automatically assigned to each tube. This scannable code is unique for every study subject and does not contain elements of their demographics. The code will identify study subjects throughout documentation and evaluation and will be traceable only by the principal investigators. The biobank, located at the study coordinating center, will be monitored by the principal investigator.

Separate samples of whole blood, red blood cells, buffy coat, plasma, and serum will be coded and stored in freezers at −80°C available at the sampling site until further analysis.

### Follow-Up

All of the described assessments will be repeated during follow-up visits at 1, 3, and 5 years. Relevant outcomes will be measured on three different domains of (i) clinical outcome (i.e., symptoms, treatment efficacy, tolerability and cost, diagnostic stability), (ii) cognition, and (iii) social functioning and recovery (Table 1).

Patients will be invited and interviewed again to assess diagnostic stability and all symptoms will be recorded. Other assessments will include all of the assessments of the inclusion phase, any change in living situation and functioning of the patient, performance on neuro- and social cognition tests, function and disability, perceived stigma, components of metabolic disturbances, and all-cause mortality. Number of relapses, referral to the mental health care system, and re-hospitalization will be recorded.

Based on the experience of a large cohort study in our region (57), several contact information (at least three) were gathered in the time of inclusion. Along with a routine synchronization with entry of the referral mental hospital of the province, this process was found to be the best solution for the best possible rate of retention.

### Data Analysis

Primary outcomes will be measured in two categories, symptom remission, including cognitive performance, and functional remission. The analysis will be performed to relate risk/resilience factors to the outcome measures and evaluate the course of the outcome. We considered five types of outcomes: (1) dimension of psychosis (58), (2) cognitive functioning (58), (3) symptom remission (59), (4) diagnostic stability (60), and (5) functional remission (61). Based on rates reported by previous studies, a minimum number was calculated to be 374 patients. Allowing for dropouts during follow-up, we estimated the total sample size to be 500 (please see sample power analysis).

Ratings will be performed by one trained psychologist. In case of multiple raters (lie collaboration of other refereal centers in other provinces), inter-rater concordance will be examined for observer-rated tools by suitable Kappa statistics. Raw data will be checked for invalid or nonsense entries. Some raw data will be coded into composite measures and data for investigators. For example, each component of the cognitive battery might have several tasks and repetitions. These multiple results will be combined to give a summary score based on the structure of the battery. A similar process is also applicable to other measures. Double-checking of data entry is performed by a trained psychologist who is not involved in patient inclusion. Digital data are checked weekly and compared to paper record, as well as any discrepancy between test results. Disagreements are then flagged and resolved.

Descriptive statistics using tabulations and graphical methods will be performed to achieve the objective I, after double-checking data entry, data cleaning, and checking for consistency between different types of measurements. Neuropsychological test scores will be converted to domain z scores for correlational analysis to determine predictors of decline or reserve. For other questionnaires, there are predefined values for interpretation. After identifying the distribution of the data, kappa coefficients, correlations, multilevel regression, multidimensional analysis, cluster analysis, and longitudinal trajectory analysis will be used.

The magnitude of the difference between risk and resilience factors will be evaluated by comparing standardized mean differences to achieve objective II. The relationship between risk and resilience factors will be assessed by logistic regression (for binary outcomes) and multivariable linear regressions (for continuous normally distributed outcomes). Appropriate longitudinal data methods will be used for the assessment of disease progression from baseline first episode status to disease at follow-up in years 1, 3, and 5. Multivariable models will determine risk factors that predict cognitive, personal, and clinical outcomes, when appropriate. Multilevel analysis to compare disease progression will also be considered.

We will perform a cluster analysis to achieve objective III. The hierarchical cluster method will be applied to identify the number of clusters for each outcome and seed points for a k-means cluster analysis. As an example, neurocognitive subtypes will be compared on baseline clinical and socio-demographic variables by conducting Chi-square analyses or Kruskal Wallis tests on gender, age, social support, educational level, substance use, schizophrenia spectrum disorder diagnosis, symptom severity, dosage of antipsychotic medication, and adverse life events.

We will define predictive models for objective IV to assess the predictive value of (using the previous example) cognition on symptomatic and functional outcomes at follow-up. Separate clinically and statistically meaningful trajectories will be identified using censored normal group-based trajectory modeling (62) and evaluated for model classification accuracy. Predicted trajectories will be evaluated using a random-effect ordinal regression analysis.

*Power calculation:* In a recent study [(63); Submitted] in the Dutch population, we observed three symptoms trajectories of positive symptoms with a frequency of 67.8, 23.3, and 8.9%, with a range of significantly associated predictors of effect size (odds ratio), range 1.2–1.8, obtained through multinominal regression models. Translating these observations to the ARAS study consisting of 500 subjects, we expected to observe three groups of patients' clinical trajectories, respectively consisting of 340 (0.68^*^500), 115 (0,23^*^500), and 45 subjects. Give the study includes three-time points of T0, T3, and T5, we consider a simple repeated data scenario to estimate an expected power of estimate: assuming b/a ratio of 3, a = 0.05, beta 0.20, one main predictor of two levels partialized on the three trajectories (outcome levels: disease course) and thus a 3^*^2 design consisting of 6 groups, and 3 covariables, using *F*-test for ANCOVA for repeated measures analysis; we will have a power of 0.63 to detect small effect (*f* ) size of 0.15 at a *p*-value of 0.05, 0.86 to detect a medium *f* of 0.25; and full power to detect a large *f* of 0.40 at a *p*-value of 10^−6^. Thus, ARAS study will offer sufficient power to detect factors with medium and large effects on various multi-levels analyses. We used G^*^power ver 3 for power calculation.

## Discussion

The ARAS cohort study will have additive value by characterizing SSD and other psychotic disorders in a population living in the Middle East. Given the extensive differences between various aspects of life in Middle Eastern and Western countries, it is highly expected that the ARAS will offer a novel understanding of disease mechanisms and outcomes in SSD. However, the most important novel aspect is the study population. Several differences are hypothetically present when available studies are compared to our population. Iranian patients may not only have a slightly different genetic susceptibility, especially concerning population-specific rare genetic variants compared to other countries, but they live in quite different environmental conditions for social support, public stressors, religion, culture, eating habits, inequality, lifestyle, and the other risk/resilience factors that may influence the course of the disorder. This diversity provides valuable and rich untouched research material. A first example is illicit drugs (64), as there is an obvious difference between Iran, with opioids as the most common drug, and European countries, where cannabis use is more prevalent. Another example would be different lifestyles and diet-intakes that putatively influence the emergence of metabolic disturbances in patients with psychotic disorders (65). The ARAS study is also the first study on the quality of care for patients with SSD in Iran. Gathering information on a wide variety of treatments, the project *per se* will improve the quality of care for participating patients and can stand as the first step in this pathway.

The project might face several limitations that come with longitudinal natural cohort studies. Data collection may be hampered by the duration of the diagnostic battery and arrangements. Training of the interviewers and use of standardized procedures for data collection is expected to contribute to a low proportion of missing data, and no imputation is being planned. Patients may be lost to follow-up because of e.g., distance, stigma, and lack of insight. Incomplete data from illiterate patients is another challenge; however, illiteracy will not interfere with many aspects of evaluations, such as the diagnostic interview and several parts of the cognitive battery as well as the expected outcome. On the other hand, improvement in the care provided during the project might increase the cooperation of patients and their families.

In conclusion, the ARAS cohort will yield significant academic- (research and education) and care-related achievements. This study will provide novel data about risk and resilience factors for SSD. The National health care system will benefit from reliable data on care for patients with SSD. ARAS data and experience will have value both in being a useful model for other parts of this region and in the expansion of the currently available knowledge. Eventually, ARAS will help to optimize healthcare for people with SSD and improve the quality of life of this vulnerable group of people.

## Data Availability Statement

The original contributions presented in the study are included in the article/supplementary material, further inquiries can be directed to the corresponding author/s.

## Author Contributions

SF drafted the manuscript and it was modified by by all authors. RB, BA, and SF conceived the study and were major contributors to writing the manuscript. WV, MG, AM, MS, and AS-K contributed to the design and study protocol. All authors read and approved the final manuscript.

## Conflict of Interest

The authors declare that the research was conducted in the absence of any commercial or financial relationships that could be construed as a potential conflict of interest.
